# Supplementary material for: Dynalign II: common secondary structure prediction for RNA homologs with domain insertions
Source: Nucleic Acids Res. 2014 Nov 21;42(22):13939–48. doi: 10.1093/nar/gku1172 (PMC4267632; doi:10.1093/nar/gku1172)
Supplement: SUPPLEMENTARY DATA [file supp_gku1172_nar-02021-z-2014-File012.zip › manual/Overview/Repository_Organization.html]

RNAstructure Installation and Overview: Repository Organization


|  |  |  |
| --- | --- | --- |
|  | RNAstructure Installation and Overview Repository Organization | - Contents - Index |
| Code Directories Directory \| Description; RNA\_class \| A group of related object wrappers that encapsulate most back end code for easy use; src \| Location of most back end code GUI Directories Directory \| Description; RNAstructure\_java\_interface \| Cross-platform Java interface capable of running on Linux, Macintosh, or Windows operating systems; RNAstructure\_windows\_interface \| GUI writen natively (using MFC) for Windows operating systems Text Interface Directories Directory \| Description; AllSub \| Generate a group of suboptimal structures; bifold \| Bimolecular folding; bipartition \| Bimolecular partition function for predicting pair probabilities; CircleCompare \| Compare two structures for the same sequence, with the nucleic acid backbone arranged around a circle to facilitate easy comparisons; ct2dot \| Convert CT structure notation to dot bracket structure notation; dot2ct \| Convert dot bracket structure notation to CT structure notation; DotPlots \| Creates three separate interfaces that can make different dot plot types \|; draw \| Generate Postscript images of secondary structures; DuplexFold \| Bimolecular folding without intramolecular pairs; dynalign \| Predict the lowest free energy structure common to two sequences; efn2 \| Predict folding free energy change of a given structure; EnsembleEnergy \| Predict the energy of a structure ensemble.; fold \| Single sequence secondary structure prediction by free energy minimization; MaxExpect \| Secondary structure prediction by maximizing expected accuracy; multilign \| Predict the lowest free energy structure common to more than two sequences; napss \| Use NMR data to improve the prediction of an RNA secondary structure; oligoscreen \| Screen a set of oligonuceotides for hybridization stability and self-structure; oligowalk \| Calculate thermodynamic features of sense-antisense hybridization and predict free energy changes of oligo binding to target RNA; PARTS \| Predict pair probabilities for folding of two sequences to a common structure; pfunction \| Partition function calculation of base pairing probabilities; ProbablePair \| Generate structures composed of only highly probable pairs as determined by a threshold; ProbKnot \| Predict pseudoknots for a single sequence; refold \| Refold a previously folded structure; RemovePseudoknots \| Remove pseudoknots from a structure; RNAstructure\_windows\_text\_interfaces \| Text interfaces specifically for Windows; scorer \| Calculate sensitivity and positive predictive value for two structures being compared; stochastic \| Stochastic sampling from the Boltzman ensemble; TurboFold \| Predict the lowest free energy structure common to more than two sequences, using base pairing probabilities. Resource Directories Directory \| Description; data\_tables \| Location of thermodynamic parameters; exe \| Holds executables for text interfaces, GUIs, and any libraries; tests \| Project tests for each text interface Help Directories Directory \| Description; examples \| Sample input files; manual \| Help and some usage documentation Essential Top-Level Files File Name \| Description; compiler.h \| Compiler and compilation flags, macros, and definitions; gpl.txt \| The GPL license under which the RNAstructure software package falls; library\_defines.h \| Names for the libraries the repository builds, defined by operating system; Makefile \| The main Makefile which builds most of the repository modules; Version.txt \| Contains the most recent version number and release date for the repository | | |
| Visit The Mathews Lab RNAstructure Page for updates and latest information. | | |
